# Supplemental parenteral nutrition improves patient outcomes after esophageal cancer surgery: A single-center randomized controlled study

**DOI:** 10.1097/MD.0000000000031893

**Published:** 2022-11-25

**Authors:** Bindong Xu, Hao Chen, Qiang Zhang, Pengfei Chen

**Affiliations:** a Department of Thoracic and Cardiovascular Surgery of the Affiliated Hospital of Putian University, Putian, Fujian, China.

**Keywords:** esophageal cancer, immune function, inflammatory response, nutritional status, supplemental parenteral nutrition

## Abstract

We investigated the effect of supplemental parenteral nutrition comprising parenteral nutrition (PN) and enteral nutrition (EN) on the postoperative nutritional status, immune function, and inflammatory response of patients with esophageal cancer.

**Methods:**

Seventy-two patients with esophageal cancer were divided into the experimental group (PN + EN group; n = 36) and control group (total EN [TEN] group; n = 36). In the PN + EN group, EN and PN were administered on postoperative days 4 to 8. In the TEN group, EN was initiated on postoperative days 1 to 8. Changes in the nutritional status, immune function, and inflammatory indices were compared between groups.

**Results:**

Before surgery, the prealbumin (PA) values of both groups were lower than normal, and the C3, C4, and C-reactive protein (CRP) levels were above normal. The IgA, IgG, IgM, CD3, CD4, and CD4/CD8 levels were lower than normal, and the CD8 level was increased. On postoperative day 1, the PA levels of both groups decreased (*P* > .05), C3, C4, and CRP levels increased, and IgA, IgG, IgM, CD3, CD4, and CD4/CD8 decreased to values noted before surgery. On postoperative day 7, PA levels of the PN + EN group were significantly higher than those of the TEN group (*P* < .05). The CRP level of the PN + EN group was significantly lower than that of the TEN group (*P* < .05). IgA, IgG, and CD4 were significantly higher in the PN + EN group than in the TEN group (*P* < .05).

**Conclusion:**

Supplemental parenteral nutrition for perioperative esophageal cancer patients can maintain the optimal nutritional status, improve immune function, and reduce the inflammatory stress response.

## 1. Introduction

Esophageal cancer is 1 of the most common gastrointestinal malignancies encountered in clinical practice,^[1]^ and its mortality and incidence are ranked fourth and sixth, respectively, among all malignant tumors in China.^[2]^ When esophageal cancer is treated, most patients have middle-stage or late-stage disease, and they often experience trouble swallowing or have varying degrees of dysphagia. Therefore, these patients experience different degrees of negative nitrogen balance, immunosuppression, and stress before surgery. Surgery is 1 of the most effective methods of treating esophageal cancer, but the trauma and postoperative stress associated with surgery and anesthesia can further exacerbate the conditions of these patients. Rational nutritional support can improve the clinical outcomes of critically ill patients.^[3,4]^ When the gastrointestinal function is normal, enteral nutrition (EN) support is preferred.^[3]^ However, in clinical practice, because of gastrointestinal intolerance of transient blockade, it is challenging to implement early postoperative EN for most patients with esophageal cancer. Simple EN support usually requires 5 to 7 days for the target amount of nutritional support to be achieved.^[5,6]^ Therefore, this study focused on the effects of supplemental parenteral nutrition (SPN) on the postoperative nutritional status, immune function, and inflammatory response of patients with esophageal cancer.

## 2. Materials and methods

### 2.1. Patient characteristics

Eligibility criteria for patients were as follows: malignant tumor (excluding small cell carcinoma) observed using a biopsy with an electronic gastroscope; no basal metabolic disease (such as hyperthyroidism); no hematopoietic system disease; no preoperative radiotherapy and/or chemotherapy; no use of hormones or immunosuppressants; no splenectomy and/or thymectomy; and no autoimmune diseases. The exclusion criteria were as follows: perioperative albumin infusion; intraoperative and/or postoperative bleeding exceeding 800 mL and receipt of a blood transfusion; nonresectable disease; refusal to participate in the study; and dropping out of the study after partial study completion.

**Figure 1.**
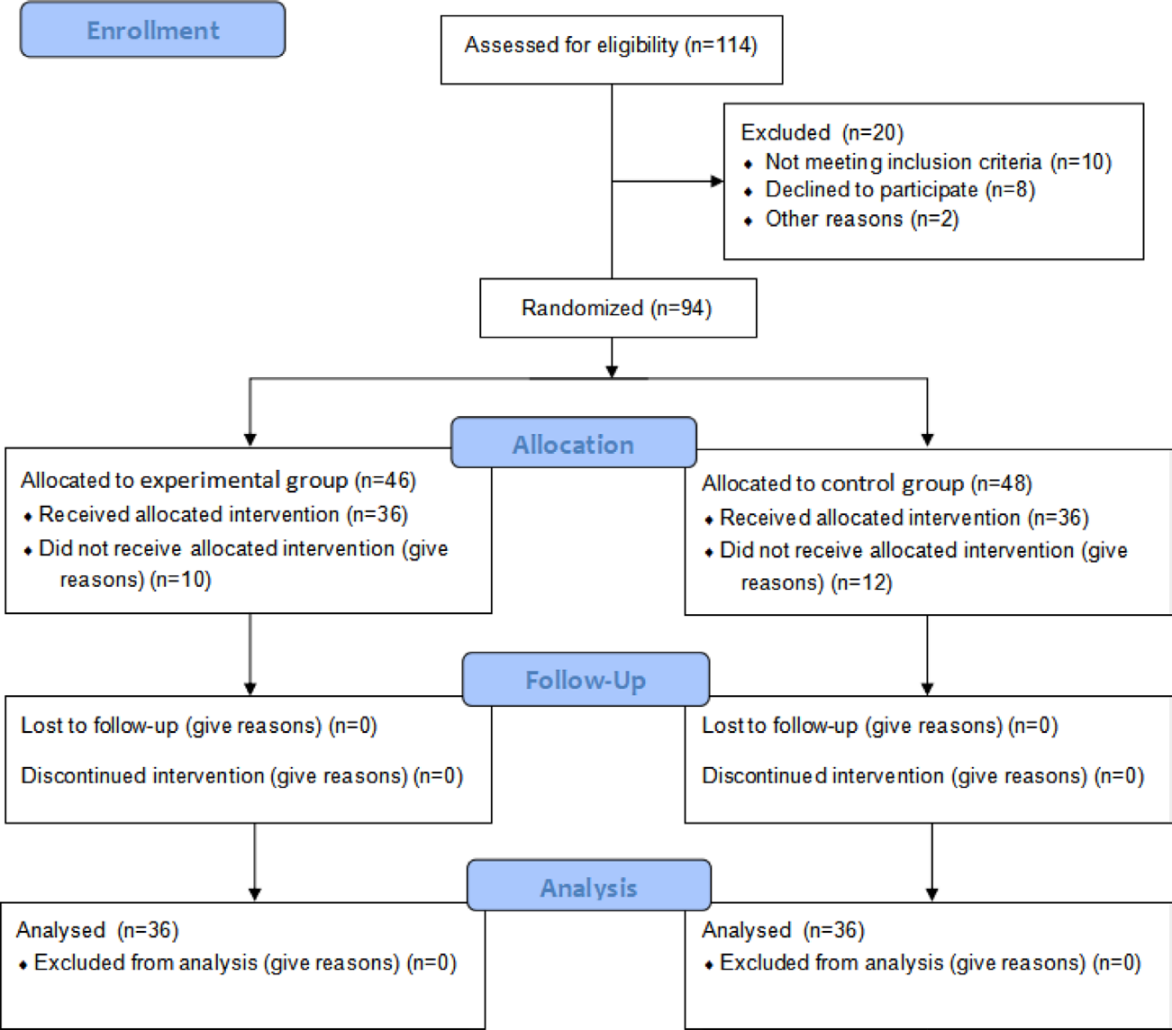
Patient flow chart including the number of screenings performed, the number of participants enrolled, the number of patients who dropped out, and final number of patients.

From June 2018 to June 2020, 72 patients with esophageal cancer treated at our department who met the aforementioned conditions were selected. There were 43 men and 29 women with an average age of 65.7 years (±6.84 years; range, 48–79 years). The enrolled patients or relatives provided signed informed consent. Using the New Drug Data statistical processing software random table, participants were randomly divided into the experimental group (PN + EN group) or control group (total enteral nutrition [TEN] group); there were 36 participants in each group (Fig. 1). Postoperative pathological diagnosis of all esophageal carcinomas was squamous cell carcinoma, and all esophagogastric junction carcinomas were adenocarcinomas. Postoperative pathological staging was performed according to the 2009 International Union Against Cancer and American Joint Committee on Cancer criteria.^[7]^ This study was approved by the ethics committee of our hospital. No statistically significant differences existed between the groups in terms of sex, age, tumor site, surgical anastomosis site, postoperative pathological stage, tumor differentiation, and nutritional score (according to Nutritional Risk Screening 2002) (Table 1).

**Table 1:** Comparison of the 2 patient groups.

Characteristics	Experimental group	Control group	χ^2^/*t* value	*P*-value
Sex, male/female (n)	23/13	20/16	0.520	.47
Age (years)	66.19 ± 6.911	65.28 ± 6.839	0.566	.57
Tumor site (n)			0.592	.90
Upper chest	6	5		
Middle chest	18	16		
Lower chest	10	12		
Esophagogastric junction	2	3		
Surgical anastomosis site (n)			0.575	.75
Neck anastomosis	22	19		
Supra-aortic anastomosis	12	14		
Subaortic anastomosis	2	3		
Postoperative pathological staging (n)			2.898	.41
Stage I	4	3		
Stage II	12	9		
Stage III	13	20		
Stage IVA	7	4		
Degree of tumor differentiation (n)			0.940	.63
Well-differentiated	9	12		
Moderately differentiated	20	16		
Poorly differentiated	7	8		
Nutrition score (Nutritional Risk Screening 2002)	3.11 ± 1.369	3.75 ± 1.713	1.634	.13

## 3. Methods

### 3.1. Study design

This study was a 2-group, parallel, single-center, randomized, controlled, assessor-blinded trial.

### 3.2. Subjects

The study protocol was approved by the Ethics Committee of our university (approval number 202039). All patients enrolled in the study provided written informed consent.

### 3.3. Sample size determination

After preliminary experiments, the average prealbumin (PA) values of the control group and the experiment group were 150 mg/L (±50 mg/L) and 180 mg/L (±40 mg/L), respectively. The pooled standard deviation of the group was 50. The clinically significant difference between groups was 5, with an *α* of 0.05 and *β* of 0.2. The experimental group and control group were matched 1:1, and the estimated loss to follow-up rate was 10%. The final sample size of the experimental group was 36 cases, and the final sample size of the control group was 36 cases.

### 3.4. Randomization and blinding

Using the New Drug DataThe participants did not know which group they were in, but the researchers did.

### 3.5. Parenteral nutrition formula

Parenteral nutrition was infused separately through the central vein (right subclavian or right internal jugular vein) daily starting at 9:00 am via an infusion pump and continued for 20 hours. The nitrogen source was provided by a compound amino acid injection (18AA-V-SF, 250 mL: 8.06 g; Hubei Bantian Pharmaceutical Co, Ltd). Various oil and fat emulsion injections (C6-24, 250 mL; Fresenius Kabi Austria GmbH) and alanyl-glutamine injections (100 mL: 20 g; Fresenius Kabi Huarui Pharmaceutical Co, Ltd) were also administered. The formula also comprised water-soluble and fat-soluble vitamins and trace elements (10 mL; Guangdong Shixin Pharmaceutical Co, Ltd). Insulin and glucose were administered at 1 U: 4 g and adjusted according to blood sugar and electrolyte levels.

### 3.6. Enteral nutrition preparation

The EN emulsion (Rui Neng; 200 mL/bottle; energy density, 1.3 kcal/mL; Fresenius Kabi Huarui Pharmaceutical Co, Ltd) was administered through a nasoenteral nutrition tube (Fuerkai; CH12; Netherlands Nutricia Export Co, Ltd) daily starting at 9:00 am. An infusion pump and a pipeline heater controlled the dripping speed for 20 hours.

All patients in the 2 groups received EN support therapy within 48 hours after surgery, and the target total calorie count was 30 kcal/kg/day. The EN preparation group was given 260 kcal on the first postoperative day; this amount was increased by 260 to 520 kcal/day. However, in cases of abdominal distension and diarrhea, among other reasons, the calorie intake after 4 days of EN therapy did not reach 60% of the target. The TEN group (control group) continued to receive complete EN therapy, and the amount was gradually increasing according to the patient’s intestinal tolerance. Gastrointestinal symptoms were treated. The PN + EN group, beginning on day 4, the PN + EN group (experimental group) administered SPN in addition to EN therapy so that the total calories reached the target total calories.

### 3.7. Observation indicators

The following indicators of all patients were measured before surgery and on days 1 and 7 after surgery: albumin, transferrin, and PA (nutritional indicators); CD3, CD4, CD8, and CD4/CD8 (cellular immune indicators); IgA, IgG, and IgM (humoral immune indicators); and C-reactive protein (CRP) and complement C3 and C4 (inflammatory indicators).

The nutritional, inflammatory, and humoral immune indicators were detected using the turbidimetric method. Cellular immune indicators were detected using the red blood cell rosette method.

Clinical indicators included postoperative gastrointestinal function recovery time, postoperative hospital stay, postoperative complications (pulmonary infection, anastomotic leakage, incision infection, urinary tract infection, and blood system infection), and total hospitalization expenses.

### 3.8. Statistical analysis

SPSS version 20.0 software (IBM Corp., Armonk, New York, USA) was used for data analyses. Data such as age, nutritional score, and other measurements are expressed as mean ± standard deviation. The *t* test was used to perform comparisons between groups. Sex, tumor site, and other enumeration data were compared using the chi-square test. Statistical significance was set at *P* < .05.

## 4. Results

### 4.1. Compliance and security

Of the 72 patients enrolled, patient compliance was 100%. No adverse events were reported, thus demonstrating safety.

### 4.2. Intake of calories and protein in the 2 groups

Before surgery, the calorie intake and protein intake of both groups were lower than the normal values. On postoperative day 1, the calorie intake and protein intake of both patient groups did not reach 60% of the target. On postoperative day 7, the calorie intake and protein intake of both groups increased. The difference between the values of the experimental and preoperative values was statistically significant (*P* < .05), as was the difference between the values of the experimental group and those of the control group (*P* < .05) (Table 2).

**Table 2:** Comparison of the calorie intake and protein intake of the 2 patient groups.

Characteristics		Experimental group	Control group	*t* value	*P*-value
Energy target (kcal/d)	Preoperative	707 ± 65	712 ± 73	0.794	.43
	Postoperative day 1	960 ± 82	980 ± 86	−0.980	.33
	Postoperative day 7	1658 ± 356*#	1360 ± 269*	3.216	.01
Protein target (g/d)	Preoperative	41 ± 3	42 ± 3	−0.588	.56
	Postoperative day 1	52 ± 5	53 ± 4	−1.818	.08
	Postoperative day 7	82 ± 7*#	66 ± 4*	2.773	.01

* Compared with preoperative values, *P* < .05.

#Compared with the control group, *P* < .05.

### 4.3. Nutritional indicators

Before surgery, the PA values of both groups were lower than the normal value. On postoperative day 1, PA values of both groups further decreased. On postoperative day 7, the PA levels of both groups increased. The difference between the values of the experimental group and preoperative values was statistically significant (*P* < .05), as was the difference between the values of the experimental group and those of control group (*P* < .05) (Table 3).

**Table 3:** Comparison of nutritional indicators of the 2 patient groups.

Characteristics		Experimental group	Control group	*t* value	*P*-value
PA (mg/L)	Preoperative	144.86 ± 46.785	130.39 ± 50.912	1.265	.21
	Postoperative day 1	95.34 ± 37.931*	81.60 ± 37.675*	1.542	.13
	Postoperative day 7	186.70 ± 40.876*Δ#	131.62 ± 53.371*Δ	4.916	<.001
TF (g/L)	Preoperative	1.79 ± 0.480	2.05 ± 0.630	−1.981	.05
	Postoperative day 1	1.39 ± 0.372	1.46 ± 0.454	−0.778	.44
	Postoperative day 7	1.72 ± 0.326	1.85 ± 0.373	−1.577	.12
ALB (g/L)	Preoperative	34.39 ± 5.200	33.38 ± 5.279	0.823	.51
	Postoperative day 1	26.21 ± 2.655*	27.30 ± 3.320*	−1.537	.13
	Postoperative day 7	34.25 ± 4.745Δ	32.47 ± 4.510Δ	1.634	.11

* Compared with preoperative values, *P* < .05.

Δ Compared with postoperative day 1, *P* < .05.

#Compared with the control group, *P* < .05.

ALB = albumin, PA = prealbumin.

### 4.4. Humoral immune index

Before surgery, the IgA, IgG, and IgM levels of both groups were below normal. On postoperative day 1, the IgA, IgG, and IgM levels of both patient groups further decreased. On postoperative day 7, the IgA and IgG levels of the PN + EN group (experimental group) significantly increased. The difference was statistically significant compared with the preoperative values (*P* < .05) and the values of the TEN group (control group) (*P* < .05) (Table 4).

**Table 4:** Comparison of the humoral immune indexes of the 2 patient groups.

Characteristics		Experimental group	Control group	*t* value	*P*-value
IgA (g/L)	Preoperative	0.55 ± 0.449	0.47 ± 0.390	0.792	.43
	Postoperative day 1	0.48 ± 0.391	0.41 ± 0.299	0.893	.38
	Postoperative day 7	0.88 ± 0.421*Δ#	0.59 ± 0.444	2.847	.01
IgG (g/L)	Preoperative	5.16 ± 3.356	5.48 ± 3.303	−0.407	.69
	Postoperative day 1	4.21 ± 3.442	4.08 ± 2.982	0.163	.88
	Postoperative day 7	10.70 ± 4.392*Δ#	6.34 ± 4.203	4.301	<.001
IgM (g/L)	Preoperative	0.38 ± 0.324	0.35 ± 0.295	0.350	.73
	Postoperative day 1	0.32 ± 0.256	0.30 ± 0.265	0.294	.77
	Postoperative day 7	0.36 ± 0.225	0.36 ± 0.262	−0.092	.93

* Compared with preoperative values, *P* < .05.

Δ Compared with postoperative day 1, *P* < .05.

#Compared with the control group, *P* < .05.

### 4.5. Cellular immune indicators

Before surgery, the CD3 and CD4 values and CD4-to-CD8 ratios of both groups were lower than the normal values. The CD8 values were higher than normal. On postoperative day 1, the CD3 and CD4 values and CD4-to-CD8 ratios of both patient groups decreased, whereas the CD8 value increased. On postoperative day 7, the CD3 and CD4 values and CD4-to-D8 ratios of the PN + EN group (experimental group) increased, whereas the CD8 values decreased. Compared with the preoperative values, the difference was statistically significant (*P* < .05). The increased CD4 level was significantly higher in the PN + EN group (experimenal group) than in the TEN group (control group) (*P* < .05) (Table 5).

**Table 5:** Comparison of cellular immune indexes of both patient groups.

Characteristics		Experimental group	Control group	*t* value	*P* value
CD3 (%)	Preoperative	40.86 ± 12.497	42.31 ± 8.085	−0.582	.56
	Postoperative day 1	31.75 ± 11.809*	28.03 ± 9.294*	1.486	.14
	Postoperative day 7	45.92 ± 14.308Δ	40.69 ± 11.104Δ	1.730	.09
CD4 (%)	Preoperative	21.69 ± 8.535	24.11 ± 12.094	−0.980	.33
	Postoperative day 1	14.19 ± 5.392*	16.06 ± 9.084*	−1.057	.29
	Postoperative day 7	27.06 ± 10.529*Δ#	21.14 ± 7.302Δ	2.771	.01
CD8 (%)	Preoperative	51.25 ± 6.644	49.14 ± 6.525	1.360	.18
	Postoperative day 1	59.03 ± 8.140*	56.39 ± 7.423*	1.437	.16
	Postoperative day 7	43.64 ± 11.339*Δ	47.83 ± 8.087Δ	−1.807	.08
CD4/CD8	Preoperative	0.42 ± 0.157	0.50 ± 0.283	−1.448	.15
	Postoperative day 1	0.24 ± 0.092*	0.29 ± 0.176*	−1.426	.16
	Postoperative day 7	0.66 ± 0.334*Δ#	0.46 ± 0.181Δ	3.217	.01

* Compared with preoperative values, *P* < .05.

Δ Compared with 1 day after surgery, *P* < .05.

#Compared with the control group, *P* < .05.

### 4.6. Inflammatory indicators

Before surgery, the C3, C4, and CRP levels of both groups were higher than the normal values. On postoperative day 1, the C3, C4, and CRP levels of both patient groups increased. On postoperative day 7, the CRP level of the PN + EN group (experimental group) significantly decreased. The differences were statistically significant compared with those before surgery (*P* < .05) and compared with those of the TEN group (control group) (*P* < .05) (Table 6).

**Table 6:** Comparison of inflammatory indexes of both patient groups.

Characteristics		Experimental group	Control group	*t* value	*P* value
C3 (g/L)	Preoperative	2.16 ± 0.998	2.21 ± 0.855	−0.232	.82
	Postoperative day 1	3.33 ± 0.513*	3.55 ± 0.748*	−1.435	.16
	Postoperative day 7	3.28 ± 0.369	3.31 ± 0.522	−0.310	.76
C4 (g/L)	Preoperative	0.64 ± 0.334	0.78 ± 0.355	−1.791	.08
	Postoperative day 1	0.92 ± 0.203*	1.04 ± 0.332*	−1.760	.08
	Postoperative day 7	0.78 ± 0.120	0.82 ± 0.363Δ	−0.607	.55
CRP (mg/L)	Preoperative	18.35 ± 9.235	20.59 ± 7.724	−1.114	.27
	Postoperative day 1	24.06 ± 15.107	26.60 ± 15.316	−0.708	.48
	Postoperative day 7	8.90 ± 7.556*Δ#	16.24 ± 13.528Δ	−2.842	.01

* Compared with preoperative values, *P* < .05.

Δ Compared with postoperative day 1, *P* < .05.

#Compared with the control group, *P* < .05.

### 4.7. Clinical indicators

The postoperative gastrointestinal function recovery time of the experimental group was shorter than that of the control group (*P* < .05). The incidence of postoperative pulmonary infection of the experimental group was lower than that of the control group (*P* < .05). The anastomotic leakage incidence of the experimental group was lower than that of the control group (*P* < .05). The postoperative hospital stay of the experimental group was lower than that of the control group (*P* < .05). The total hospitalization cost of the experimental group was higher than that of the control group (*P* < .05) (Table 7).

**Table 7:** Comparison of clinical indicators of both patient groups.

Characteristics	Experimental group	Control group	*t* value	*P* value
Postoperative complications (cases)			15.881	.01
Lung infection	8	16	4.000	.05
Anastomotic fistula	2	9	5.258	.02
Incision infection	2	1	0.354	.55
Urinary tract infection	1	2	0.354	.55
Blood system infection	2	1	0.354	.55
Catheter-related infection	2	1	0.354	.55
Postoperative hospital stay (d)	14.17 ± 4.790	18.47 ± 6.340	−3.251	.01
Total hospitalization cost (yuan)	71,261.94 ± 11,503.500	65,226.81 ± 10,106.431	2.365	.02

## 5. Discussion

Nutritional support therapy, including EN and PN, is essential after esophageal cancer surgery. Relevant guidelines for nutritional support treatment indicate that EN support is preferred when the gastrointestinal function is normal. However, gastrointestinal insufficiency may occur because of the stress response after esophageal cancer surgery, resulting in EN intolerance. When the EN instillation rate is too fast and severe complications, such as aspiration pneumonia, occur because of acid reflux, pure EN may not meet the energy and nutritional needs of patients, especially those of elderly patients. A long-term lack of energy after esophageal cancer surgery can cause excessive consumption of normal tissues and interfere with normal anabolism, thereby affecting the prognosis.^[8]^

SPN refers to mixed nutritional support treatment whereby energy and protein requirements are supplemented by PN when the energy supply of EN is insufficient. In this study, the preoperative PA levels of both patient groups were lower than the normal value, suggesting different degrees of malnutrition before esophageal cancer surgery. On postoperative day 1, the PA levels further decreased, indicating that the trauma of surgery led to metabolic disorders in the body, thus affecting cell anabolism and aggravating malnutrition. However, on postoperative day 7, the PA level of the experimental group recovered significantly, exceeding normal values. The difference was statistically significant compared to that of the control group. This is because, when using EN to maintain the intestinal mucosal barrier integrity, PN supplementation can meet the energy and protein requirements of the body after surgery. Sufficient nutrient substrates can promote protein synthesis in the body and improve the negative nitrogen balance, thereby improving the nutritional status of patients with esophageal cancer after surgery and reducing the occurrence of postoperative complications.^[9]^ SPN may be more conducive to the synthesis of visceral proteins in the body.

SPN may be beneficial for reducing the postoperative stress response in esophageal cancer patients. The CRP level is a sensitive indicator of the stress state. When the body is in a state of immune stress, the synthesis of complements C3 and C4 increases accordingly.^[10]^ In this study, the CRP levels of both patient groups were higher than the normal values, suggesting that patients with esophageal cancer were in a state of stress before surgery. On postoperative day 1, the CRP, C3, and C4 levels significantly increased, suggesting that surgery trauma further aggravated the stress response. On postoperative day 7, the CRP level of the experimental group decreased significantly, and the difference was statistically significant between groups.

The incidence of infection after surgery for esophageal cancer is related to the nutritional status and immune status of the body.^[11]^ Glutamine, which is added to SPN, is an energy source for intestinal mucosal epithelial cells. Glutamine is essential for maintaining the structure and integrity of the intestinal mucosa because it provides sufficient nitrogen sources for the body and reduces the stress response.^[12]^ Additionally, the milk fat in SPN contains omega-3 polyunsaturated fatty acids, which can also reduce the inflammatory response; furthermore, SPN allows the digestive tract to rest, thereby reducing its inflammatory response.^[13]^

The preoperative IgA, IgG, and IgM levels of both patient groups were lower than normal, indicating that the patients with esophageal cancer had different degrees of humoral immune dysfunction before surgery. On postoperative day 7, the IgA and IgG levels of the experimental group increased significantly, with a statistically significant difference between groups. The reason for this could be related to the presence of glutamine in SPN. Glutamine, as the precursor of protein synthesis, is also a vital transporter of nitrogen between organs and tissues, and it can promote the synthesis of B lymphocytes and secretion of related antibodies.^[14]^ SPN may improve the humoral immune function of esophageal cancer patients after surgery.

The results of this study suggest that the CD3 and CD4 levels and CD4-to-CD8 ratios of both patient groups were lower than the normal values, indicating that cellular immune function was low in patients with esophageal cancer before surgery. On postoperative day 7, the CD4 count of the experimental group increased significantly, with a statistically significant difference observed between groups. SPN may enhance the cellular immune function of patients with esophageal cancer after surgery. The mechanism may be that glutamine, which is added to SPN, can improve the physiological function of immune cells, such as lymphocytes and neutrophils, in the body.^[14]^

### 5.1. Study limitations

This study had some limitatons. For example, this was a single-center study. The total number of enrolled cases was relatively small, and there was a lack of long-term follow-up. Therefore, the results must be further validated. This research should be extended to other hospitals, and a statistical analysis of the 5-year postsurgical survival rate should be performed.

## 6. Conclusion

For patients with esophageal cancer, when the energy supply fails to reach 60% of the target demand on postoperative day 4, supplemental PN support provided immediately on postoperative day 1 effectively maintains the nutritional status. Although improving the immune function and reducing the inflammatory stress response can increase the total cost of hospitalization, supplemental PN support can reduce the occurrence of postoperative complications and facilitate patient recovery.

## Acknowledgments

We thank Guozhong Huang (Department of Cardiothoracic Surgery, The Affiliated Hospital of Putian University, Putian Fujian, China) for assistance with editing this manuscript.

## Author contributions

Study conception and design: Bindong Xu and Hao Chen. Data collection: Bindong Xu, Hao Chen, Qiang Zhang and Pengfei Chen. Analysis and interpretation of results: Bindong Xu, Hao Chen and Qiang Zhang. Manuscript preparation: Bindong Xu and Hao Chen. All authors reviewed the results and approved the final version of the manuscript.

**Conceptualization:** Bindong Xu.

**Data curation:** Bindong Xu.

**Formal analysis:** Bindong Xu.

**Funding acquisition:** Pengfei Chen.

**Investigation:** Pengfei Chen.

**Methodology:** Pengfei Chen.

**Project administration:** Pengfei Chen.

**Resources:** Pengfei Chen.

**Software:** Qiang Zhang.

**Supervision:** Qiang Zhang.

**Validation:** Qiang Zhang.

**Visualization:** Qiang Zhang.

**Writing – original draft:** Hao Chen.

**Writing – review & editing:** Hao Chen.
